# Shear-Assisted Production of Few-Layer Boron Nitride Nanosheets by Supercritical CO_2_ Exfoliation and Its Use for Thermally Conductive Epoxy Composites

**DOI:** 10.1038/s41598-017-18149-5

**Published:** 2017-12-19

**Authors:** Xiaojuan Tian, Yun Li, Zhuo Chen, Qi Li, Liqiang Hou, Jiaye Wu, Yushu Tang, Yongfeng Li

**Affiliations:** State Key Laboratory of Heavy Oil Processing, China University of Petroleum, Beijing, Changping 102249 P. R. China

## Abstract

Boron nitride nanosheets (BNNS) hold the similar two-dimensional structure as graphene and unique properties complementary to graphene, which makes it attractive in application ranging from electronics to energy storage. The exfoliation of boron nitride (BN) still remains challenge and hinders the applications of BNNS. In this work, the preparation of BNNS has been realized by a shear-assisted supercritical CO_2_ exfoliation process, during which supercritical CO_2_ intercalates and diffuses between boron nitride layers, and then the exfoliation of BN layers is obtained in the rapid depressurization process by overcoming the van der Waals forces. Our results indicate that the bulk boron nitride has been successfully exfoliated into thin nanosheets with an average 6 layers. It is found that the produced BNNS is well-dispersed in isopropyl alcohol (IPA) with a higher extinction coefficient compared with the bulk BN. Moreover, the BNNS/epoxy composite used as thermal interface materials has been prepared. The introduction of BNNS results in a 313% enhancement in thermal conductivity. Our results demonstrate that BNNS produced by supercritical CO_2_ exfoliation show great potential applications for heat dissipation of high efficiency electronics.

## Introduction

Two-dimensional materials (TDM) have received tremendous attention since the rise of graphene^1^, due to their outstanding electronic, mechanical, and thermal properties^2,3^. Recent developments of graphene, hexagonal boron nitride (h-BN), carbon nitride and transition metal dichalcogenides have made great achievements^4^. Compared with graphene, few-layered hexagonal boron nitride nanosheets (BNNS) has a wide band gap (5–6 eV) and excellent thermal stability^5–7^. The extraordinary properties make it a promising candidate in electronic devices, energy storage devices and high-performance composites^8^. While both thermal and electrical conductivities of graphene are quite high, the BNNS possess excellent thermal conductivity and electronic insulation, thus having great potential as thermal interface materials for heat dissipation of high performance electronics^9–11^.

Preparation of BNNS with high efficiency and large scale is significant for its wide applications. However, boron nitride is more difficult to be intercalated and exfoliated than graphite because of its partially ionic interlayer bonds^12^. Up to now, scaled few-layered h-BN nanosheets are mostly prepared by either ball milling or liquid-phase exfoliation^13–17^. In terms of liquid-phase exfoliation method, the dispersion of pristine BN in isopropanol is subjected to sonication, consuming high energy/time and producing a large number of organic waste^18–22^. When it comes to the ball milling method, it damages the structure of h-BN, resulting in a great deal of defects and degrading the properties of BNNS^23^. Recently, a scalable synthesis of BNNS using gas exfoliation of bulk h-BN in liquid N_2_ is reported^2^. The process involves an expansion of h-BN at 800 °C and a following rapid quench at −196 °C, which is complicated and energy consuming.

Therefore, it is particularly necessary to find a moderate, eco-friendly and large scale chemical engineering process to fabricate excellent BNNS. In recent years, supercritical fluids have been proved to be feasible to prepare graphene^24–28^, owing to their good permeability, low viscosity and high diffusivity. Among them, supercritical CO_2_ is widely explored, because it is cheap, green and nontoxic^29,30^. Supercritical CO_2_ has been proved to intercalate and diffuse between graphite layers and exfoliate graphite into graphene nanosheets with the help of rapid depressurization^31^. It is reported that TDM such as BN, MoS_2_ and WS_2_ can be exfoliated using ultrasound assisted supercritical CO_2_^28,32^. However, the application of ultrasound is complicated, and time-consuming. Meanwhile, the physical and chemical properties and applications of the products are under investigated in the previous reports.

In our work, we have applied a shear-assisted supercritical CO_2_ exfoliation method to prepare few-layer BNNS, which presents a promising method for exfoliating layered materials even with strong interlayer bonds^12^. The rotating shear can make precursors and supercritical CO_2_ evenly dispersed, in which supercritical CO_2_ are able to diffuse into the layered structures. Upon rapid depressurization, the CO_2_ expands to gaseous state and further breaks the bonds to form mono- or few-layer BNNS^33^. The magnetic stirring motor can strike a side of bulk BN intercalated supercritical CO_2_ toward the direction of the BN surface, which extremely contributes to exfoliate ultra-thick BN into few-layer BNNS. The coupled effect of supercritical CO_2_ cooperated with shear is favorable for the exfoliation with high efficiency. The morphology and thickness of the produced BNNS have been characterized by electron microscope and atomic force microscope, which indicates that the supercritical CO_2_ exfoliated bulk h-BN successfully, resulting in few-layer nanosheets. X-ray diffraction (XRD) and Raman spectra show that the structure of the as-prepared BNNS is maintained well after the exfoliation process. Compared with bulk BN, the BNNS show better dispersibility in isopropanol with stronger absorption spectrum and higher extinction coefficient. Moreover, the as-prepared BNNS were applied as fillers in epoxy resin to prepare highly thermally conductive composites to further explore the thermal properties and applications of BNNS. The thermal conductivity of composites filled by BNNS with a high aspect ratio caused by exfoliation is much higher than that of pristine BN based composites. Experimental studies have shown that the 20 wt% BNNS results in a thermal conductivity enhancement (TCE) as high as 313%, which makes it a promising candidate as thermal interface materials^34,35^.

## Results and Discussion

### Formation mechanism

**Figure 1:**
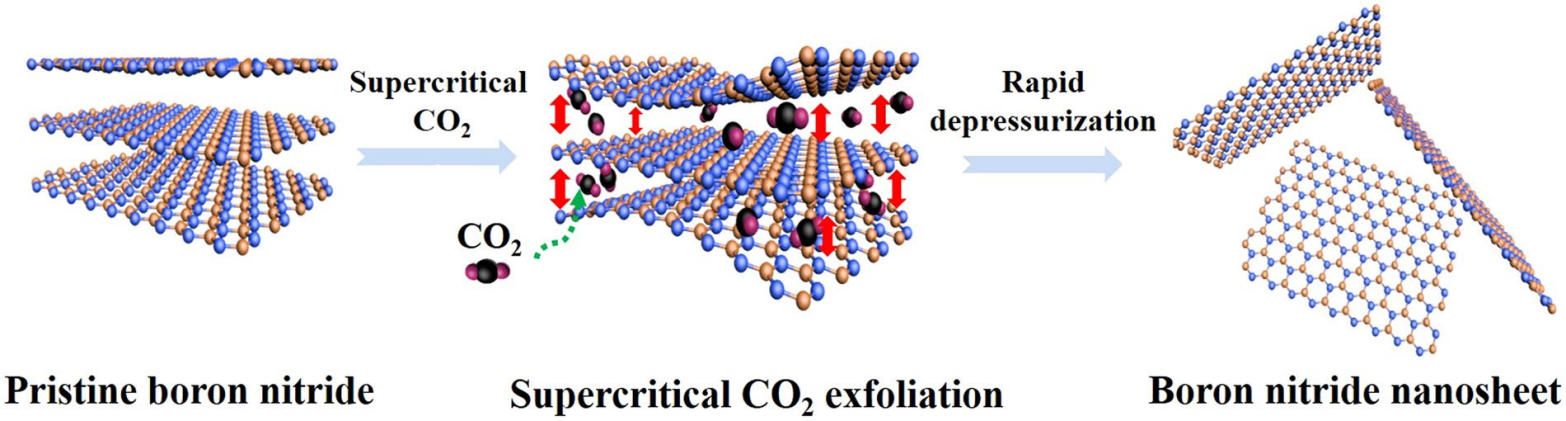
Scheme of BNNS synthesis by shear-assisted supercritical CO_2_ exfoliation.

The schematic illustration of bulk BN exfoliation by supercritical CO_2_ is shown in Fig. 1. Theoretically, boron nitride could be exfoliated by overcoming its van der Waals forces between layers. In our work, we propose a feasible mechanism for exfoliation of bulk BN using a shear mixer coupled with supercritical CO_2_ and rapid depressurization. Firstly, the shear force is produced by large velocity gradient of the supercritical CO_2_ fluid under high shear speed. With its high permeability, high diffusivity and zero interface force, supercritical CO_2_ intercalate between BN layers and weaken the van der Waals forces. The shear force helps to exfoliate the intercalated BN. Secondly, cavitation and collision proposed in the previous literature contribute to the exfoliation, too^36,37^. Cavities produced under rotating fluid of high speed grew and bubbles imploded, which produces tensile stress to promote the exfoliation of BN layers. Besides shear-assisted supercritical fluid system, the ultrasonic-assisted exfoliation was reported according to the previous literature^38–40^. In terms of ultrasonic-assisted system, the cavitation is triggered when the “negative” pressure during the rarefaction phase of the ultrasound wave is adequately large to interrupt the liquid in ultrasonic-assisted supercritical CO_2_/waterborne system. The both cavitation can act on the surfaces and sides of the particles to make cracks and promote the intercalation of supercritical CO_2_ molecules, which eventually exfoliates the bulk particles into few-layer nanosheets. Collision of particles happens frequently under high shear speed to promote exfoliation process. Last but not least, there is rapid depressurization at the end of exfoliation. CO_2_ transformed from supercritical fluid phase to gas phase during rapid depressurization accompanying a huge expansion of volume. The CO_2_, which is intercalated between layers, overcomes the van der Waals forces during the expansion process and achieves the exfoliation of bulk BN layers^24,29,30,33^.

### Characterization of boron nitride nanosheets and pristine boron nitride

**Figure 2:**
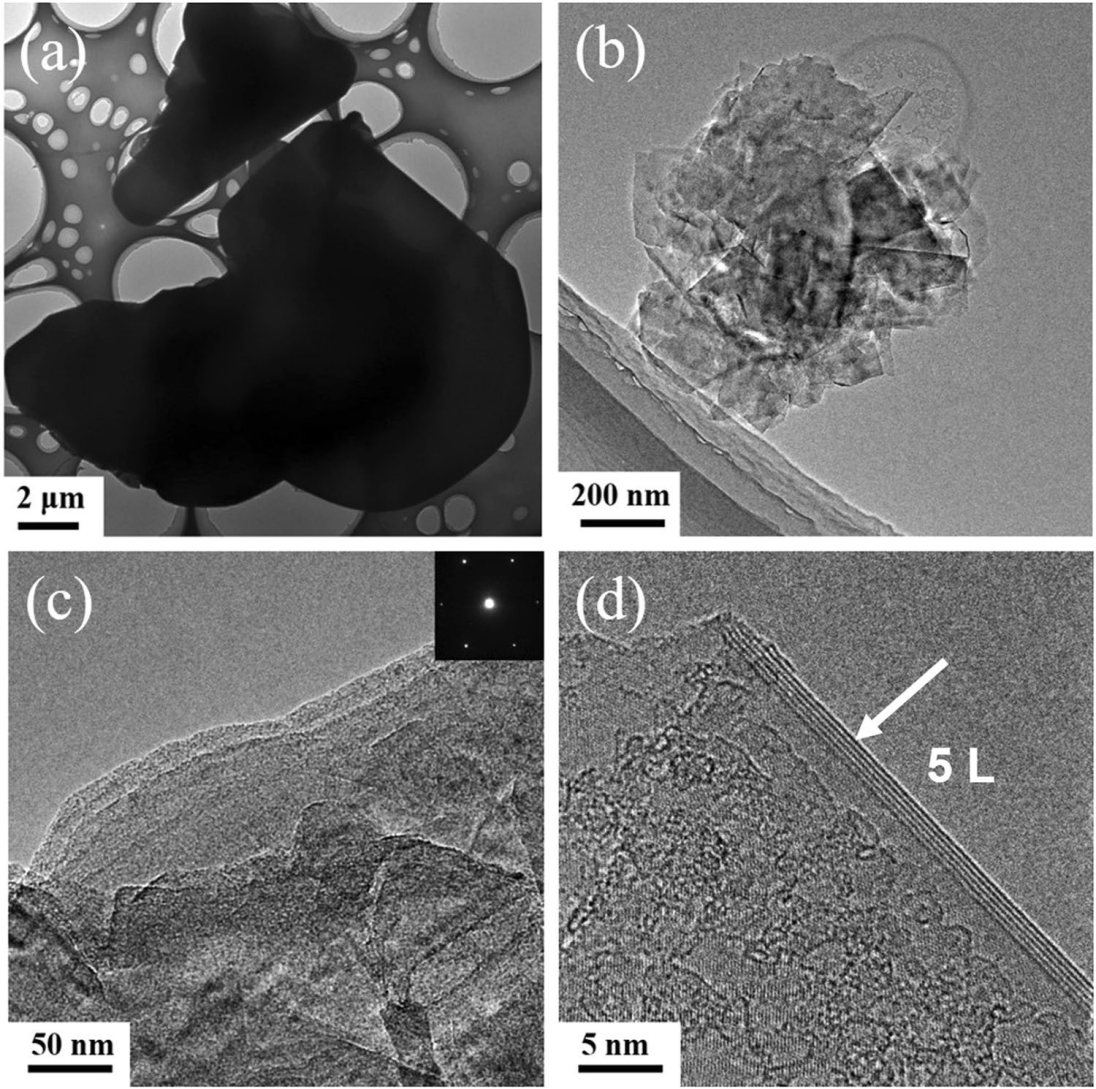
(**a**,**b**) TEM images of bulk BN and BNNS, respectively. (**c**) TEM image of BNNS, the inset of the selected-area electron diffraction pattern (**d**) HRTEM image of the edge of BNNS.

Transmission electron microscopy (TEM) was applied to characterize the morphology of samples before and after the supercritical CO_2_ exfoliation. According to the TEM images (Fig. 2a and b), it is noticeable that most of the pristine h-BN are thick flakes, with lateral size ranging from several to tens micrometers. In contrast to the bulk h-BN particles, the produced BNNS have a relatively smaller size, thinner thickness and a nanosheet-like morphology, revealing the efficient exfoliation of the bulk BN by supercritical CO_2_. Moreover, TEM images (Fig. 2c and d) of BNNS further exhibit the thickness and layers of the products to prove the effectiveness of the exfoliation method, compared with TEM images of pristine BN (Fig. 2a and Fig. S2a and b). Figure 2b and c shows that the BNNS are so ultrathin and transparent that the top layers are passed through by the electron beam to see the bottom layers. The high-resolution TEM (HRTEM) image (Fig. 2d) shows BNNS with 5 layers. The electron diffraction pattern (the inset in Fig. 2c) suggests that the BNNS has the typical six-fold symmetry, which indicates that the supercritical CO_2_ exfoliation reserves the hexagonal structure of BN well.

**Figure 3:**
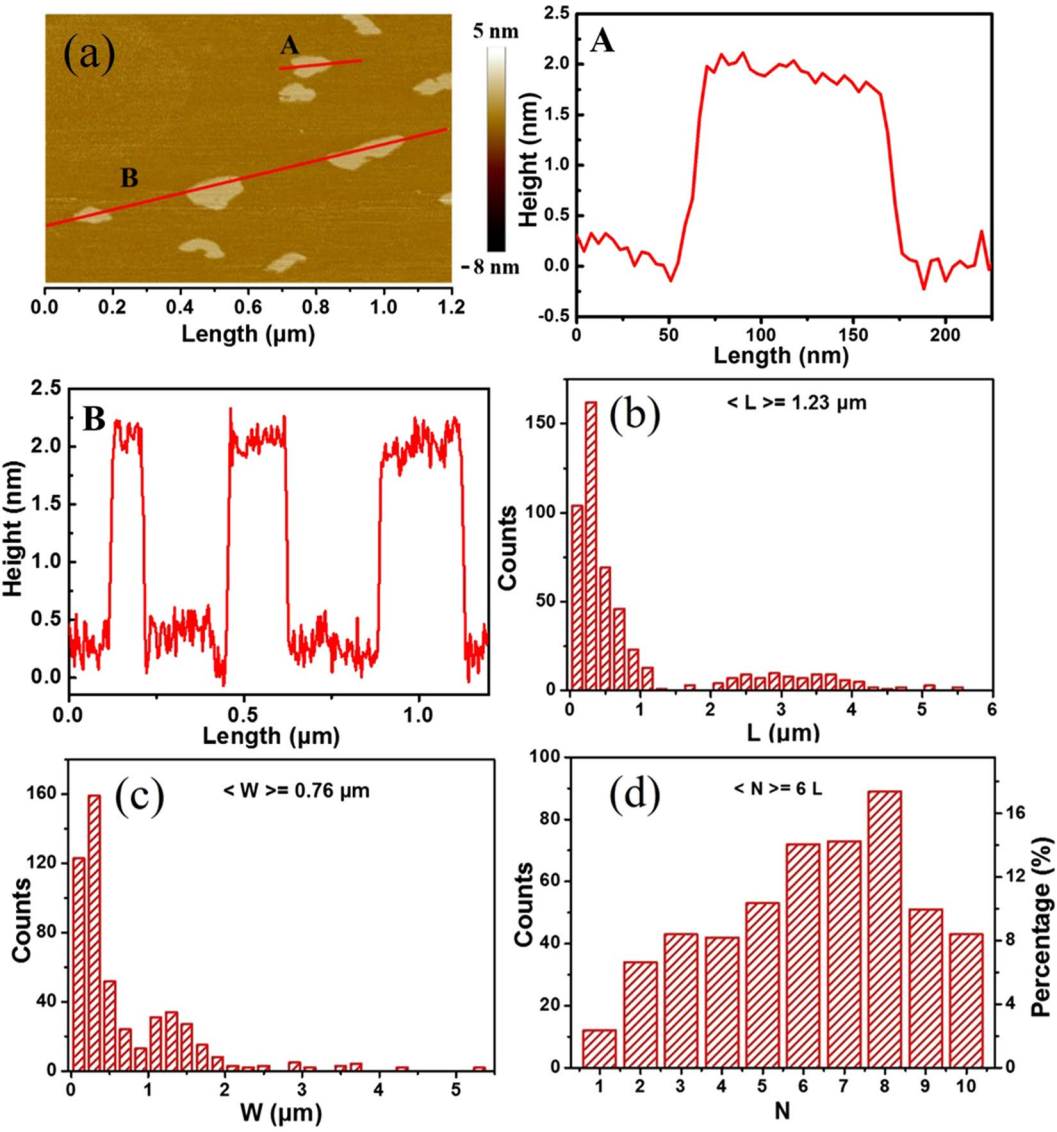
(**a**) An AFM image and their corresponding line-scan profile of BNNS (indicated by A and B), L denotes layer. (**b**,**c** and **d**) Statistical analysis of flake size for BNNS. L, W and N are the flake length, width and layers of BNNS, respectively.

Furthermore, we applied atomic force microscope (AFM) to estimate the thickness and layers of the prepared BNNS (Fig. 3a). The samples were prepared by evaporating a drop of BNNS/IPA suspension BNNS around 4 layers. More AFM images were collected, as shown in Fig. S3 in supporting information, which reveals that the produced BNNS consist mostly of 5–9 atomic layers. Further, statistical analysis provides more information about the length, width and layers distributions of the products. The results are shown in Fig. 3b–d, which is the histogram of size distribution obtained by analyzing 512 pieces of BNNS through AFM measurement (Fig. S3 and Fig. 3a). The average size of length and width of the samples is 1.23 µm and 0.76 µm, respectively. The samples are mainly composed of 5–9 layers BNNSs (74%). The result of AFM further demonstrate the high efficiency of BN exfoliation by supercritical CO_2_ exfoliation.

**Figure 4:**
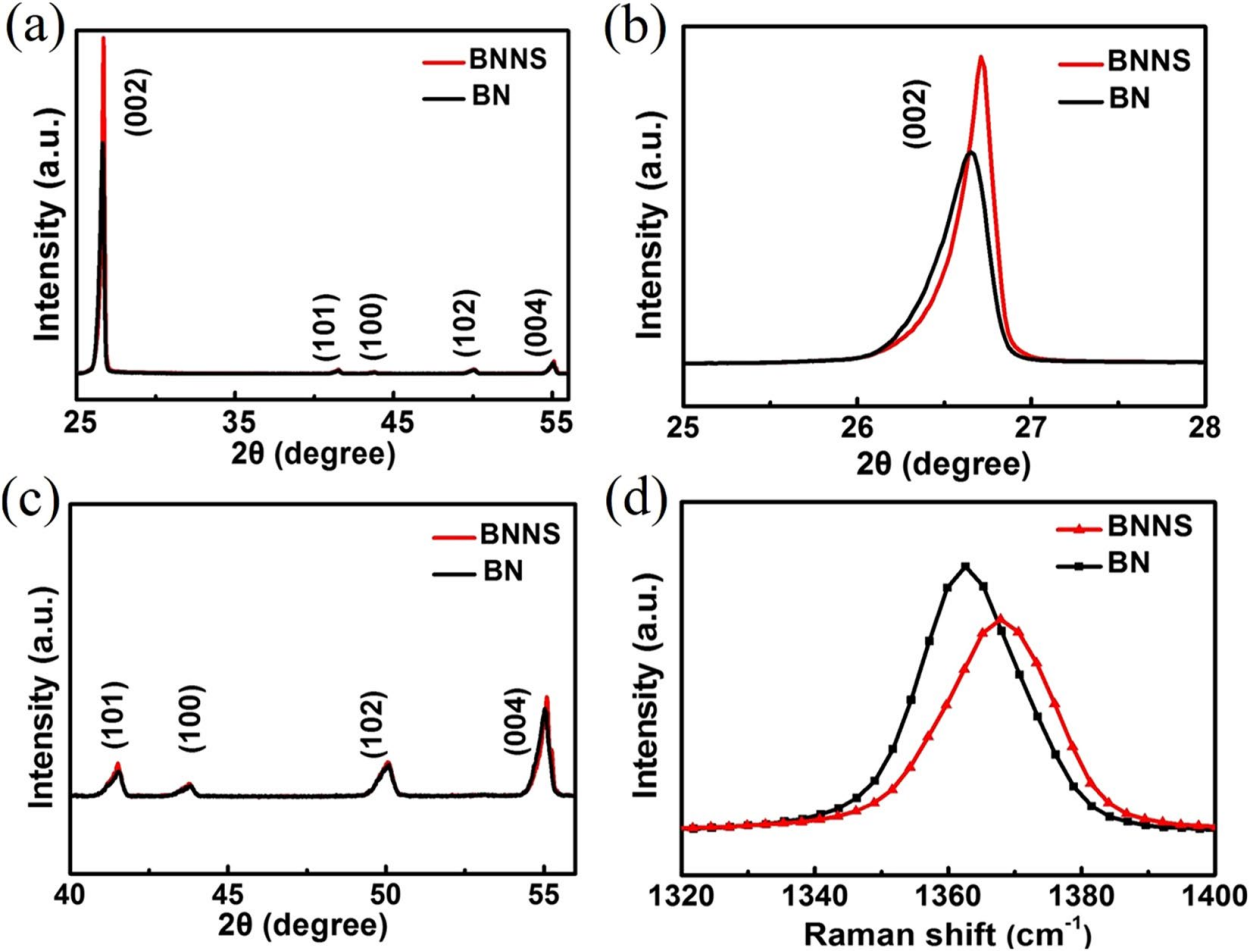
(**a**) XRD patterns of BNNS and BN. (**b**,**c**) High-resolution XRD patterns of BNNS and BN. (**d**) Raman spectra of BNNS and BN.

Figure 4a–c show the XRD pattern of bulk BN and BNNS carried out using XRD with CuKα radiation (1.5418 Å) to further investigate the crystalline structure before and after the supercritical CO_2_ exfoliation. Figure 4a brings to light a logical hexagonal structure of the h-BN with lattice constants of a = 0.251 nm and c = 0.662 nm (JCPDS card 34-0421). As shown in Fig. 4b, the intensity of the (002) diffraction peak of as-prepared BNNS is obviously larger than that of bulk BN powders, meanwhile, with the intensity of other diffraction peaks [(100), (101), (102) and (004)] unchanged (Fig. 4c). This result indicates that the bulk BN powders are exfoliated into ultrathin BNNS along the (002) plane without destroying its crystalline structure by the supercritical CO_2_, which agrees with the XRD pattern of liquid-phase exfoliated BNNS reported in previous literatures^12,41,42^.

Raman spectrum of BN and BNNS powders were conducted by using a laser wavelength (λ = 532 nm) (Fig. 4d). Raman spectra of BNNS are different from those of graphene, showing only a G band attributing to E_2g_ vibration mode due to the lack of Kohn anomaly^43^. The sharp peak of the h-BN occurs at 1366.49 cm^−1^, owing to the E_2g_ vibration mode of h-BN. Compared with the bulk BN, the G band frequency of BNNS is upshifted to 1368.65 cm^−1^. The G band upshift reveals that the bulk BN powders are exfoliated to thinner flakes, which results in a stronger in-plane strain and lower interlayer interaction^2,44^. In addition, the full widths at half-maximum (FWHM) of G band of BNNS, which is 19.72 cm^−1^, is wider than that of the bulk BN powder (12.79 cm^−1^). The upshifted FWHM and weakening peak intensity also reveal that the bulk BN powders are exfoliated to thinner flakes, which agrees with the relevant literature^22,44,45^.

**Figure 5:**
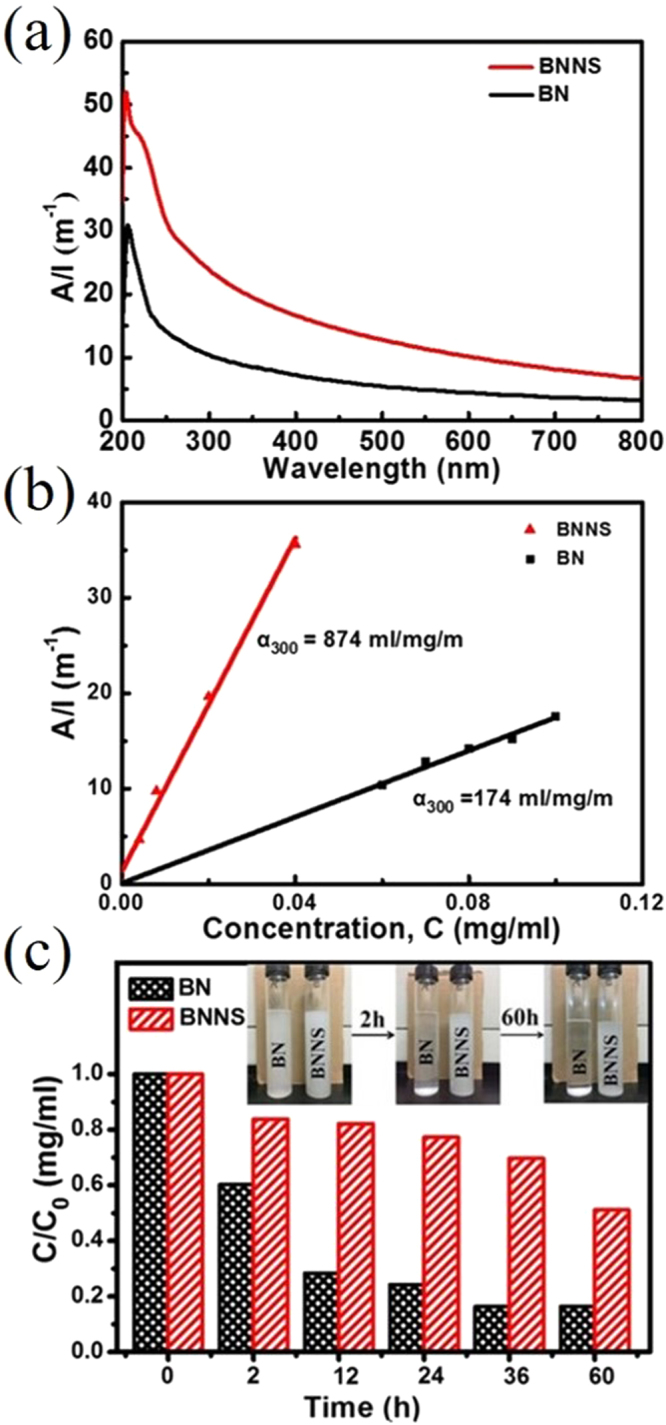
(**a**) Absorbance spectra of dispersions of BNNS and BN (0.04 mg/ml). (**b**) Lambert-Beer plots for BNNS and BN dispersions. (**c**) The ratio of the concentration of supernatant to the initial concentration of dispersion as a function of standing times. The inset showing photographs of BNNS dispersions.

Moreover, the dispersion of BNNS were prepared and characterized. We sonicated the bulk BN and as-prepared BNNS powders in IPA, respectively, to prepare homogeneous dispersion. After sonication, the two dispersions were centrifuged at 1500 rpm about 45 min. The supernatant was decanted and diluted to different concentrations. Figure 5a shows UV/Vis absorption spectrum of BN/IPA and BNNS/IPA dispersions under the same concentrations (0.04 mg/ml). It is obvious that the absorbance of BNNS/IPA is much higher than that of BN/IPA, which is attributed to the size reduction, resulting in low spectral scattering beyond the UV region and much weaker Tyndall effect^46^. In addition, there are the shoulder structures around 220 nm, which are probably attributed to a bond exciton caused by vacancies, suggesting that these crystals have mild defects^47^. According to Fig. S4a and b, we can obtain the absorbance of BN/IPA and BNNS/IPA at 300 nm, which are linearly fitted into a straight line (Fig. 5b). As shown in Fig. 5b, the solutions obey the Lambert–Beer’s law as evidenced by the linear relation between absorbance and concentration in the range of concentrations between 0.004 and 0.04 mg/mL (BNNS/IPA), which allows the calculation of the extinction coefficients (*α*_300_)^46,48^. The extinction coefficient of BNNS/IPA dispersion is higher than that of BN/IPA dispersion (Fig. 5b). The inset of Fig. 5c shows the pictures of BNNS/IPA and BN/IPA dispersions with standing time. The dispersions were prepared by sonication at the same concentration following by settling down. It is observed that the pristine BN powders in the solution precipitated on the bottom in several minutes, leaving a transparent solution, which is obviously due to the large lateral sizes and thick layers of the bulk BN precursor. Comparatively, the BNNS/IPA dispersion is much more stable. However, the slight decrease of concentration in BNNS dispersions indicate that the reassembling in the products still remains a challenge. The reassemble issues could be addressed by adding surfactant as in the previous literature^36^. The supernatant of the dispersions were collected and characterized by UV/Vis spectrum with time. And the concentration of the as-collected supernatant were calculated. Figure 5c shows the change in ratio of the supernatant concentrations to the initial concentration over time. Being consistent with what is observed in the picture, the BNNS/IPA dispersion is much more stable than BN/IPA dispersion. Around 70% BN have precipitated when dispersion were standing for 12 h, which is further increased to 80% at 60 h. After the supercritical CO_2_ exfoliation, only 20% BNNS flakes precipitate until 24 h and 51% BNNS flakes still remain stable in the dispersion after 60 h. The improved dispersibility of BNNS prepared by supercritical CO_2_ exfoliation is useful for its further chemical treatments and applications.

Due to its outstanding thermal conductivity and electrical insulation, boron nitride is a promising candidate as thermal interface materials for heat dissipation of high performance electronics. Also, we prepared BNNS/epoxy composites and measured their thermal conductivity. The composite we prepared is for applications as thermal grease, which is used to eliminate air gaps or spaces (which act as thermal insulator) from the interface area so as to maximize heat transfer. Thermal grease should be evenly spread over the entire processor surface area with good spreadability^49,50^, as shown in Fig. S7. Thus, the content of filler is determined by considering both the thermal conductivity and spreadability.

**Figure 6:**
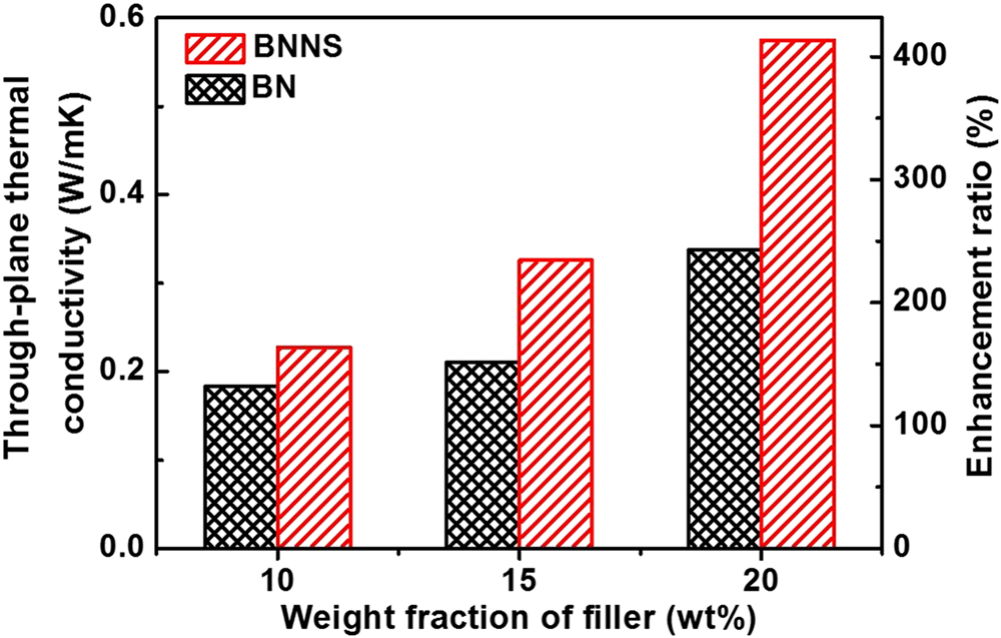
Through-plane thermal conductivities of BNNS and BN based composites.

Figure 6 reveals the through-plane thermal conductivities of the composites with fillers amount varying from 10 wt% to 20 wt%, respectively. As expected, through-plane thermal conductivities increase with the increment of BN content since the thermal conduction path is determined by the BN linkages. The thermal conductivity of the neat epoxy resin is about 0.14 W m^−1^ K^−1^. Compared with BN based composites, BNNS/epoxy composites show significantly higher through-plane thermal conductivities. The improved thermal performance of BNNS is not surprised considering the increased aspect ratio (L/T, where L is the average length of BNNS and T is the average thickness of BNNS) of exfoliated flakes. The thickness of flakes is greatly reduced with the supercritical CO_2_ exfoliation, resulting in a high ratio of lateral size to thickness. At a higher aspect ratio, the thermally conductive linkages could form at a lower filler concentration. Based on the morphology characterizations before, the aspect ratio of produced BNNS is as high as 400. As a reference, the aspect ratio of graphene fillers in thermal interface materials is 200–350^51,52^. The high aspect ratio results a through-plane thermal conductivity increasing continually from 0.23 to 0.58 W m^−1^ K^−1^ while the BNNS content increases from 10 wt% to 20 wt%. The through-plane thermal conductivity of composite based on BNNS (BNNS content, 20 wt %) with no chemical treatment are mostly in the range of 0.2 W m^−1^ K^−1^ to 0.45 W m^−1^ K^−1^ according to the previous literature^53–56^. The high thermal conductivity of 0.58 W m^−1^ K^−1^ of our work indicates the successful and efficient exfoliation of BN by shear-assisted supercritical CO_2_ fluid. The TCE is calculated as the ratio of thermal conductivity of composites to that of raw epoxy resin. The 20 wt% BNNS results in a TCE as high as 313%, showing great potential as thermal interface materials.

## Conclusion

In conclusion, we have successfully prepared single or few-layer boron nitride nanosheets by shear-assisted supercritical CO_2_ exfoliation method. Compared with widely used ball milling and liquid-phase exfoliation, supercritical CO_2_ exfoliation has the advantages of eco-friendly, save time and energy and easy to scale up. It is demonstrated that bulk boron nitride is exfoliated efficiently by the supercritical CO_2_, which is confirmed TEM and AFM measurements. Moreover, the crystalline structure of the boron nitride is maintained well after the exfoliation process, which confirmed by XRD and Raman spectrum. The produced BNNS have a superior dispersibility, which aids for the preparation of uniform BNNS based composites. Besides, the BNNS show a great potential as fillers in thermal interface materials. The BNNS/epoxy resin composites are very attractive for the thermal management of various applications, such as next-generation electronic devices, power systems, and communication equipment.

## Experimental section

### Materials

Bulk boron nitride (99%) was purchased from Liaoning DCEI Co. Ltd., China, with lateral size around 30 μm. Carbon dioxide (99.5%) was obtained from Beijing AP BAIF Gases Industry Co. Ltd., China. Isopropyl alcohol (IPA) (98%) was purchased from Tianjin Guangfu technology development Co., Ltd., China. Epoxy resin was supplied by Guangdong guanghua Sci-Tech Co., Ltd. (China).

### Preparation of boron nitride nanosheets

In terms of BNNS preparation, we have developed a viable apparatus, which mainly includes six parts including gas cylinder, chiller, pump, reactor, magnetic stirring motor and collector, as shown in Fig. S1 in supporting information. The magnetic stirring motor is made up of a 4-blade rotor. The size of blade is shown in Fig. S5. Under the high-speed rotating process, there will be two radial shear force in the vertical direction.

A certain amount of bulk BN (about 1 g) was added into a cylindrical reactor (250 mL) and heated by electric heating to 60 °C. Then, the gaseous CO_2_ was cooled into liquid under the action of chiller and transported to the reactor by adjusting the valve. The liquid CO_2_ was gasified and further pressurized up to 12 MPa by pump. When the temperature and the pressure reached to the prospective value, the magnetic stirring motor run about 1 h under a certain rotating speed (1200 rpm). High pressure and rotating speed are favorable for exfoliation of layered materials in shear-assisted supercritical CO_2_ system according to the previous literature^36^. However, ultrahigh parameters are not easy to reach for scaled-up system in industry considering cost and safety. Thus, we pick the moderate parameter to provide preliminary investigation for the possibility and performance of scaling up the shear-assisted supercritical CO_2_ exfoliation system. Finally, the vent valve was opened to maximum for rapid depressuration, and the BN powders were collected. The above steps are repeated for eight cycles to achieve a complete exfoliation. The yield of products after eight cycles is around 11.5%. The yield for each cycle is showed in Fig. S6, which exhibits that the yield originally rapidly increased but stabilized after 5 cycles.

### Fabrication of BNNS/epoxy composite

The BNNS or pristine BN was dispersed in IPA by shear mixing for 30 min, respectively. Then, the dispersion was fully mixed in IPA by sonication for 3 h. Epoxy resin was dropped into the dispersion during stirring, and the prepared mixture was kept stirring for 30 min to form a uniform dispersion. The mixture was heated in a water bath at about 85 °C for 4 h to remove most IPA. Finally, the obtained mixture was placed in a vacuum oven at about 85 °C for 5 h for complete removal of the IPA. The composite were prepared utilizing a dual asymmetric centrifuge Speed Mixer (Sienox, SIE-C200) to produce homogeneous and spreadable thermal greases. Photos of the thermal grease is added in the supporting information (Fig. S7).

### Characterizations

The morphologies and layers of the acquired BNNS, pristine BN was characterized by transmission electron microscopy (TEM, FEI Tecnai G2 F20). The length, width and height were also characterized by atomic force microscopy (AFM, Bruker Multimode 8). Structure characterizations of BNNS and bulk BN were carried out by an X-ray diffractometer (XRD, Bruker D8 Advance) and an Xplora plus Raman confocal microscope system (Horiba scientific). Optical absorption spectrum was conducted on a UV/Vis spectrometer (UV-2600, Shimadzu). The thermal conductivity of BNNS and bulk BN composites was measured on a TIM Thermal Resistance & Conductivity Measurement Apparatus (LW-9389, Long Win Science & Technology Co).

## Electronic supplementary material

Supplementary Information
