# Supplementary material for: Shear-Assisted Production of Few-Layer Boron Nitride Nanosheets by Supercritical CO2 Exfoliation and Its Use for Thermally Conductive Epoxy Composites
Source: Sci Rep. 2017 Dec 19;7:17794. doi: 10.1038/s41598-017-18149-5 (PMC5736726; doi:10.1038/s41598-017-18149-5)
Supplement: Supplementary file 1 — Supplementary Information [file 41598_2017_18149_MOESM1_ESM.doc]

**Supporting Information**

**Shear-Assisted Production of Few-Layer Boron Nitride Nanosheets by Supercritical CO_2_ Exfoliation and Its Use for Thermally Conductive Epoxy Composites**

Xiaojuan Tian^1^, ^†^, Yun Li^1, †^, Zhuo Chen^1^, Qi Li^1^, Liqiang Hou^1^, Jiaye Wu^1^, Yushu Tang^1^ and Yongfeng Li^1^, *

^1^ State Key Laboratory of Heavy Oil Processing, China University of Petroleum, Beijing, Changping 102249, P. R. China

* Corresponding author: Email: yfli@cup.edu.cn.

^†^ These authors contributed equally to this work.

**Supplementary Figures**


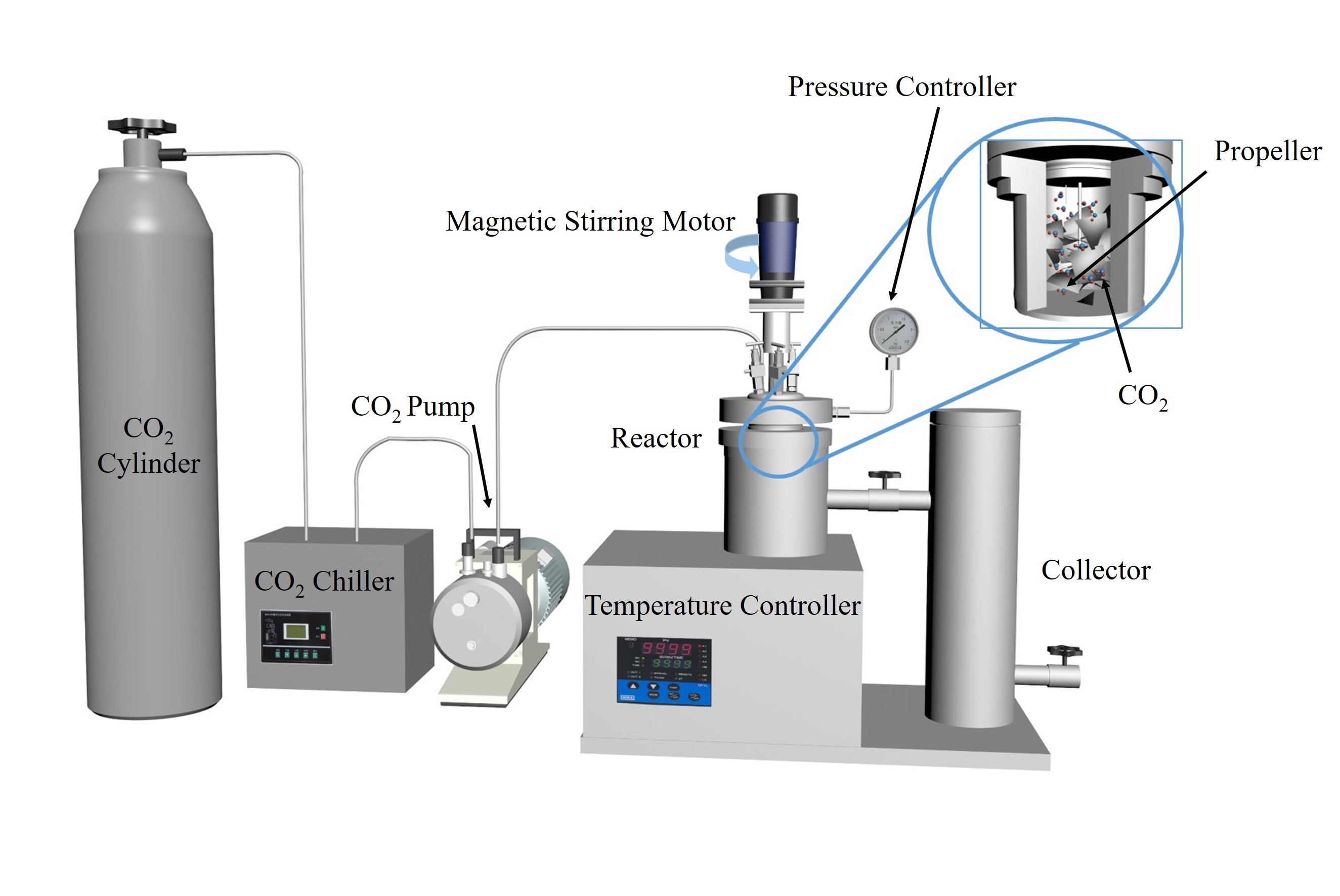


Fig. S1. Schematic illustration of experimental setup of preparing BNNS by shear-assisted supercritical CO_2_ exfoliation


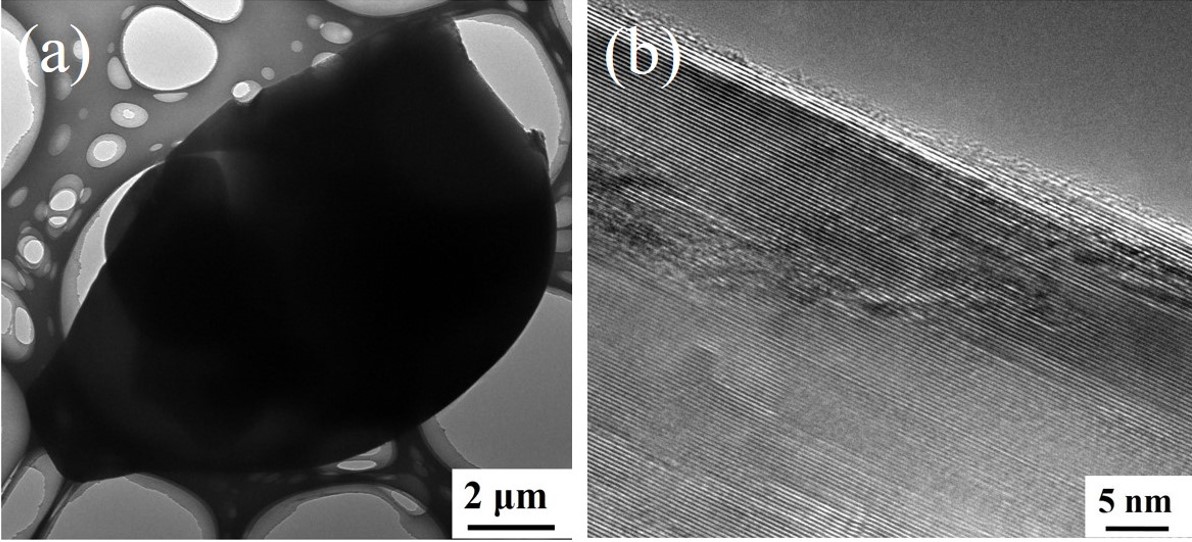


Fig. S2. (a) A TEM image of pristine BN (b) An HRTEM image of the edge of pristine BN.


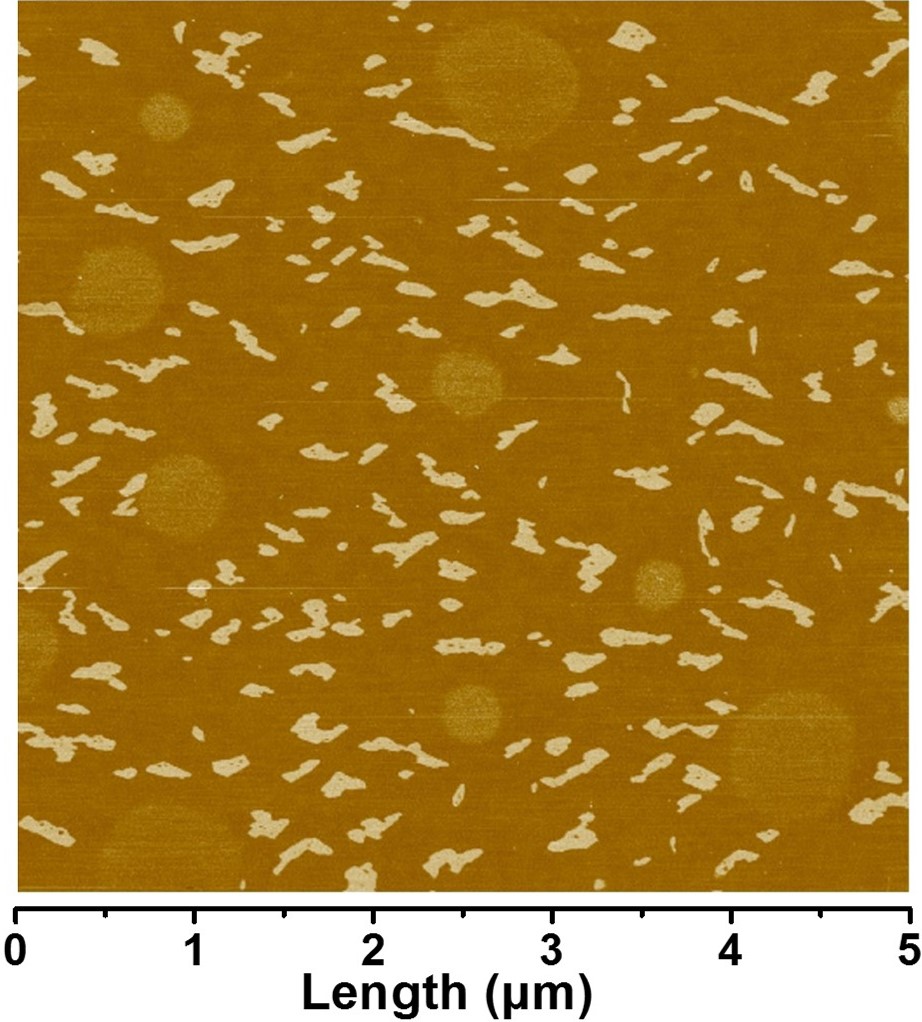


Fig. S3. An AFM image of BNNS.


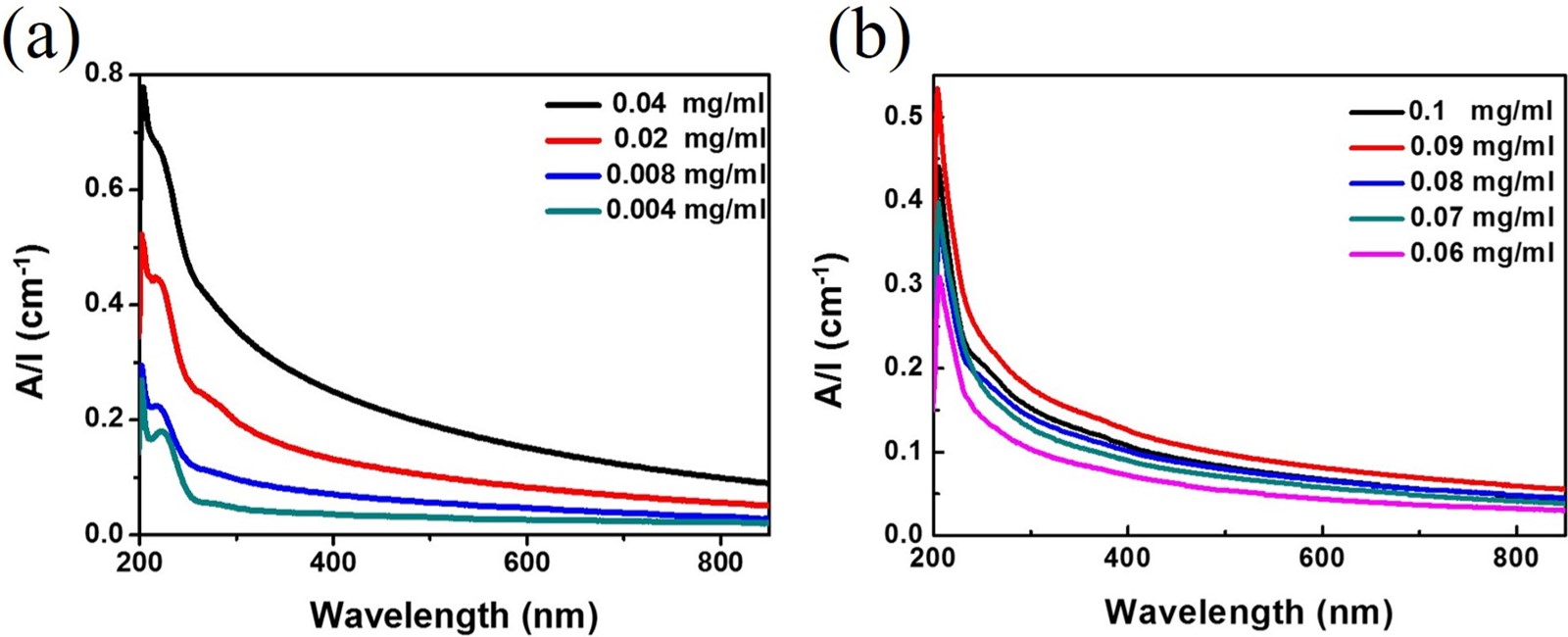


Fig. S4. Absorbance spectra of different concentrations of BNNS/IPA (a) and BN/IPA (b) dispersions.


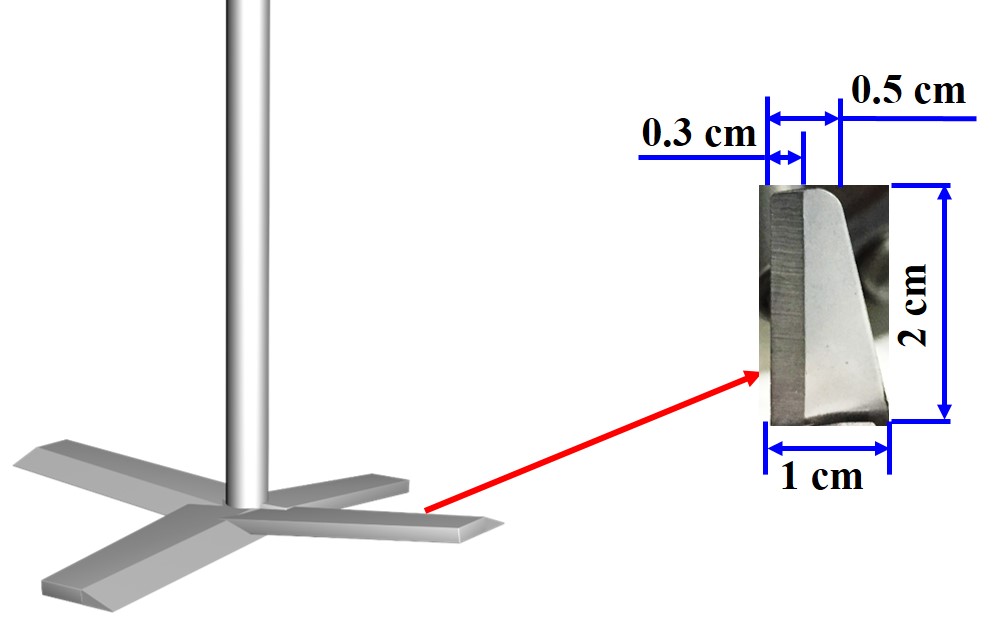


Fig. S5. The shear head.


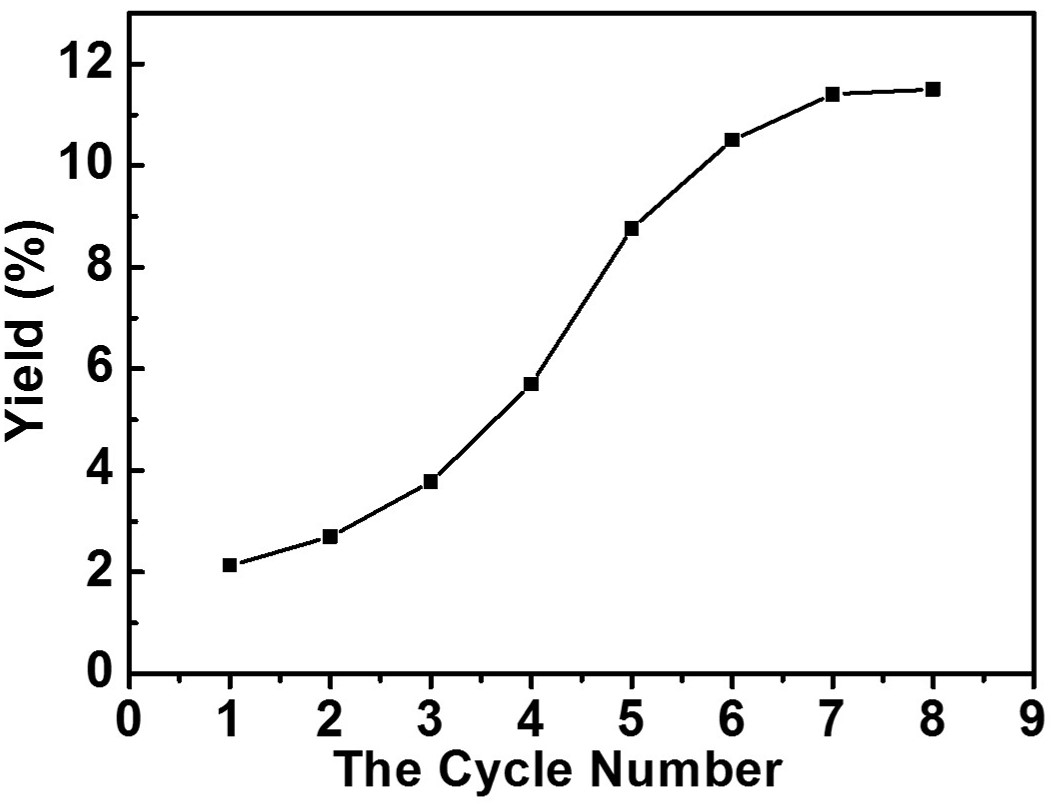


Fig. S6. The yield of the different cycle number.

1. Method of measuring exfoliation yield

The yield of products after eight cycles is around 11.5%. Bulk boron nitride is feed into the shear-assisted supercritical CO_2_ system, and collected after rapid depressurization. The collected sample is feed into the same reactor for exfoliation and collection again for a new cycle. After eight cycles, the yield of BNNS was determined by the following process. The products were stirred about 10 minutes in alcohol and sonicated for 30 minutes to achieve a homogeneous dispersion. Then, the dispersion was centrifuged at 800 rpm for 10 minutes to remove most bulk BN particles. The products in the supernatant was collected by filtration and measured as the BNNS yield.


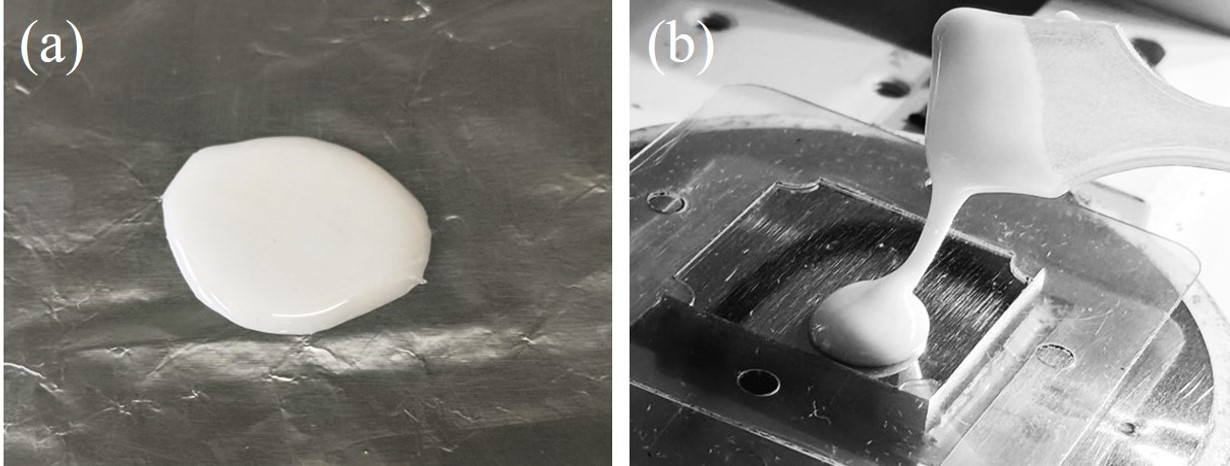


Fig. S7. The BNNS/epoxy composites.
